# Predicting Intentions of a Familiar Significant Other Beyond the Mirror Neuron System

**DOI:** 10.3389/fnbeh.2017.00155

**Published:** 2017-08-25

**Authors:** Stephanie Cacioppo, Elsa Juan, George Monteleone

**Affiliations:** ^1^Pritzker School of Medicine, Biological Science Division, Department of Psychiatry and Behavioral Neuroscience, University of Chicago Chicago, IL, United States; ^2^High-Performance Electrical NeuroImaging Laboratory, Center for Cognitive and Social Neuroscience, CCSN, University of Chicago Chicago, IL, United States; ^3^Department of Psychology, University of Geneva Geneva, Switzerland

**Keywords:** social neuroscience, mirror neuron system, embodied cognition, fMRI

## Abstract

Inferring intentions of others is one of the most intriguing issues in interpersonal interaction. Theories of embodied cognition and simulation suggest that this mechanism takes place through a direct and automatic matching process that occurs between an observed action and past actions. This process occurs via the reactivation of past self-related sensorimotor experiences within the inferior frontoparietal network (including the mirror neuron system, MNS). The working model is that the anticipatory representations of others' behaviors require internal predictive models of actions formed from pre-established, shared representations between the observer and the actor. This model suggests that observers should be better at predicting intentions performed by a familiar actor, rather than a stranger. However, little is known about the modulations of the intention brain network as a function of the familiarity between the observer and the actor. Here, we combined functional magnetic resonance imaging (fMRI) with a behavioral intention inference task, in which participants were asked to predict intentions from three types of actors: A familiar actor (their significant other), themselves (another familiar actor), and a non-familiar actor (a stranger). Our results showed that the participants were better at inferring intentions performed by familiar actors than non-familiar actors and that this better performance was associated with greater activation within and beyond the inferior frontoparietal network i.e., in brain areas related to familiarity (e.g., precuneus). In addition, and in line with Hebbian principles of neural modulations, the more the participants reported being cognitively close to their partner, the less the brain areas associated with action self-other comparison (e.g., inferior parietal lobule), attention (e.g., superior parietal lobule), recollection (hippocampus), and pair bond (ventral tegmental area, VTA) were recruited, suggesting that the more a shared mental representation has been pre-established, the more neurons show suppression in their response to the presentation of information to which they are sensitive. These results suggest that the relation of performance to the extent of neural activation during intention understanding may display differential relationships based on the cognitive domain, brain region, and the cognitive interdependence between the observer and the actor.

## Introduction

Reading mental states and inferring intentions of others plays a fundamental role in successful social interactions (Wolpert et al., 2003; Sebanz and Frith, 2004; Frith and Frith, 2006; Aglioti et al., 2008; Cacioppo et al., 2014; Rizzolatti et al., 2014; Friston and Frith, 2015; Isoda, 2016; Rizzolatti and Sinigaglia, 2016). The mechanism underlying successful intention understanding remains, however, one of the most intriguing and unresolved issues in social interactions and neuroscience (Friston and Frith, 2015). Theories on embodied cognition and simulation suggest that this mechanism underlying intention understanding takes place through “a direct and automatic matching process between an observed action and previously performed actions, via the reactivation of past self-related sensorimotor experiences” (di Pellegrino et al., 1992; Rizzolatti and Sinigaglia, 2016). Within this matching process, the inferior frontoparietal network, including the mirror neuron system (MNS), plays a critical role by facilitating the observer's understanding of actions and intentions performed by other people (Grafton, 2009; Maranesi et al., 2014; Rizzolatti and Sinigaglia, 2016). Although the activation of the MNS is not a pre-requisite to act or to understand others' actions, a growing number of studies has shown that one reads actions and infers intentions of other people by shaping one's understanding and anticipation of the environment based on one's own motor system i.e., based on the congruency with integrated templates of past self-related motor experiences (Becchio et al., 2006, 2012; Niedenthal, 2007; Rizzolatti and Sinigaglia, 2016). The working model is that the “anticipatory representations of others' behaviors require internal predictive models of actions formed from pre-established, shared representations between the observer and the actor” (Cacioppo et al., 2014). This model also suggests that one should be better at predicting intentions performed by a familiar actor, rather than a stranger (Ortigue and Bianchi-Demicheli, 2008). This model is reinforced by the psychological model of self-expansion during social relationship, which assumes that people in a significant relationship tend to expand their self, and integrate the self of their partner into a shared mental representation of the couple, rather than the individual. Initially described for trait attributes, this model now includes visuo-motor integration of the couple's motor action, intentions, and desire (Ortigue et al., 2010b; Cacioppo, 2017). Accordingly, close partners tend to develop a “transactive”, shared mental representation of their self while acting —“a mental representation that calls for cognitive interdependence and includes a structure of stored information across the two individuals” (Wegner et al., 1985; Ortigue et al., 2010b; Cacioppo et al., 2014). In dyads, cognitive interdependence calls for mechanisms underlying the concept of “inclusion of the other in the self” (IOS; as measured with the IOS scale; Aron et al., 1992). This concept is closely tied to self-expansion mechanisms, and embodied cognition (Ortigue et al., 2010b,c). Although we are all interdependent to some degree, the model of shared representation highlights “the extent to which partners may implicitly read and influence each other's perceptions of their actions, emotions, and intentions” (Cacioppo et al., 2014). Cognitive interdependence may facilitate processes underlying the prediction and anticipation of actions and intentions performed by others. Couples with high level of cognitive interdependence are expected to be more efficient and more often successful in predicting each other's actions and intentions due to their pre-established shared mental representations (Ruscher et al., 2003; Ortigue et al., 2010b; Cacioppo et al., 2014). In a prior study which involved video clips of actions performed either by the participants themselves (familiar actor), their significant other (familiar actor with whom the participants have a high IOS level), their friend (a familiar actor with whom the participants have a lower IOS level), or strangers (a non-familiar actor; Ortigue et al., 2010b,c), we, for instance, showed that participants were faster at inferring intentions performed by their significant other with whom they had a high level of IOS, than intentions of individuals with whom they had low IOS levels. Moreover, participants were as good at inferring intentions performed by their significant other than intentions performed by themselves. These findings provide an account for facilitation effects of embodied cognition and self-other expansion on intention understanding among dyads (Ortigue et al., 2010b). To test whether this facilitation effect was due to perceptual familiarity, we then tested 20 additional participants while they observed actions and inferred intentions of strangers they had seen previously (in 75% of the cases) or rarely (in 25% of the cases). No facilitation effect was found in this condition, which reinforces prior findings that suggest that the automatic facilitation effect of familiar agents on intention understanding likely occurs at “an associative, rather than a perceptual level” (Ortigue et al., 2010b).

It remains unknown, however, how the associative brain network underlying intention understanding is modulated as a function of the familiarity between the observer and the actor.

This is a critical question as we tend to spend most of our time in social settings interacting with significant others, rather than with strangers. To date, most of the studies on the neural basis of action observation and intention understanding have involved participants observing the actions performed by strangers (Jeannerod, 2001; Lewis, 2006; Grafton, 2009; Juan et al., 2013). Indeed, while neuroimaging studies have studied the neural networks sustaining familiarity (Wang and Yonelinas, 2012; Wolfe et al., 2017), person identity (Anzellotti and Caramazza, 2017), the feelings the participant experience while looking at pictures of familiar individuals (Ortigue et al., 2010a; Cacioppo et al., 2012a,b, for reviews), or the spatiotemporal brain dynamics of the priming effects of familiarity on empathy (Wang et al., 2016), or different types (friendly or hostile) of social intentions (Wang et al., 2015), the neural bases underlying how one understands actions and intentions actions performed by familiar significant others remains unclear. To address this question, we combined functional magnetic resonance imaging (fMRI) with a classic behavioral intention inference task (IIT; Ortigue et al., 2009a,b, 2010b,c), in which participants were asked to observe actions and predict intentions of the three types of actors who evoked a faciliation effect on intention understanding in our prior study (Ortigue et al., 2010b): A familiar actor with whom they had a high IOS level (their significant other i.e., partner), themselves, and a non-familiar actor (a stranger).

## Methods

### Participants

A total of 24 French-speaking, right-handed adults (14 women, 10 men; aged 18–31 years, *M* = 21.58, *SD* = 2.99), who were in long-term relationships (*M* = 27.9 months, *SD* = 20.7) with a significant other (*M*_*IOS*_ = 5.17, *SD* = 1.13), participated in the present study. All participants reported being highly satisfied with their relationship (*M* = 6, *SD* = 0.88), in love with (*M*_*passionate love scale*_ = 7.33, *SD* = 1.08; *M*_*companionate love scale*_ = 8.17, *SD* = 0.72) and committed to their significant other (*M*_commitment scale_ = 6.44, *SD* = 0.54). A full summary of all demographic information can be found in Table 1A.

**Table 1:** Demographic Information of the participants **(A)** and their feelings for their partner **(B)**.

	**Behavioral**	**fMRI**	**All subjects**	**Significance**
**A. DEMOGRAPHIC INFORMATION OF THE PARTICIPANTS**				
*n*	12	12	24	-
Males	4	6	10	-
Age	22.83 (3.61)	20.33 (1.50)	21.58 (2.99)	0.038
Anxiety	7.17 (2.21)	4.75 (2.22)	5.96 (2.49)	0.014
Depression	1.92 (1.62)	2.08 (1.88)	2.00 (1.72)	n.s.
MMS	28.75 (0.97)	29.25 (0.87)	29.00 (0.93)	n.s.
**B. FEELINGS FOR THEIR PARTNER**				
Passionate Love (PLS)	7.30 (1.23)	7.37 (0.95)	7.33 (1.08)	n.s.
Companionate Love (CLS)	8.02 (0.82)	8.33 (0.59)	8.17 (0.72)	n.s.
Commitment	6.31 (0.65)	6.58 (0.36)	6.44 (0.54)	n.s.
IOS	5.08 (1.08)	5.25 (1.22)	5.17 (1.132)	n.s.
Relationship duration (months)	34.13 (24.11)	21.63 (15.15)	27.88 (20.70)	n.s.
Loving time duration (months)	35.42 (25.31)	27.42 (7.01)	31.42 (24.59)	n.s.
Loving intensity	6.50 (0.67)	6.33 (1.07)	6.42 (0.88)	n.s.
Relationship satisfaction	5.92 (1.00)	6.08 (0.79)	6.00 (0.88)	n.s.

*All values except n and Males are averages for the sample population with standard deviations in parenthesis. Significance values reflect the comparison of the group of 12 fMRI subjects with the 12 subjects who completed only the behavioral portion of the study*.

All participants had normal or corrected-to-normal visual acuity, and no psychiatric, or neurological disorder (as ascertained by a brief anamnesis). The Institutional Review Board of the Faculty of Psychology and Educational Sciences, at the University of Geneva, approved the present protocol.

### Self-report questionnaires

Upon receipt of the participant's written consent to participate in the study, participants' anxiety and depression levels were investigated with the Hospital Anxiety and Depression (HAD) scale (Zigmond and Snaith, 1983). Mini-Mental State Examination (MMS; Folstein et al., 1975) was used in order to ascertain normal cognitive functioning. Participants' feelings and level of closeness with their significant other were assessed using the following standard questionnaires: The “Inclusion of Other in the Self scale” (IOS; Aron et al., 1992), which addresses the level of closeness and cognitive interdependence between partners on a pictorial measure showing two circles with different levels (from 1 to 7) of overlap with one another; the Passionate Love Scale (PLS; 15 questions on passionate love evaluated on a 9-point scale; Hatfield and Sprecher, 1986); the Companionate Love Scale (CLS; eight questions on companionate love evaluated on a 9-point scale; Hatfield and Sprecher, 1986); and the Commitment scale (7-point scale investigating how a participant is engaged to maintain their love relationship with their partner; adapted from Lund, 1985). Participants also answered questions regarding the duration of their relationship with their significant other, which were used in evaluating familiarity between the subject and their partner. Intensity and satisfaction of relationship were assessed with two additional questions that each participant was asked to rate on a 7 point Likert scale: “How much do you love your partner?,” and “To what extent are you satisfied with your relation?.” In the present study, IOS scores were positively correlated with love intensity, relationship satisfaction and companionate love (*p* < 0.01), and also with passionate love and commitment scores (*p* < 0.05; Table 2). Passionate love positively correlated with love intensity and relationship as well (*p* < 0.01), and negatively correlated with depression scores (*p* < 0.05). Relationship satisfaction also positively correlated with commitment (*p* < 0.05), love intensity (*p* < 0.01), and companionate love (*p* < 0.01).

**Table 2:** Correlations table among different measures.

	**Correlations**										
	**PLS**	**CLS**	**IOS**	**Commitment**	**Relationship satisfaction**	**Relationship duration**	**Loving duration**	**Loving intensity**	**HAD anxiety**	**HAD depression**	**MMS**
Age	−0.356	−0.451^*^	−0.339	−0.436^*^	−0.427^*^	0.126	0.024	−0.294	0.085	0.431^*^	−0.047
PLS		0.876^**^	0.455^*^	0.579^**^	0.791^**^	0.081	0.176	0.779^**^	−0.119	−0.504*	−0.135
CLS			0.607^**^	0.677^**^	0.831^**^	0.204	0.351	0.750^**^	−0.039	−0.339	0.018
IOS				0.433^*^	0.522^**^	0.325	0.398	0.539^**^	0.003	−0.403	0.083
Commitment					0.437^*^	0.188	0.243	0.398	−0.097	−0.304	0.388
Relationship satisfaction						−0.019	0.179	0.670^**^	0.039	−0.257	−0.053
Relationship duration							0.864^**^	0.160	0.266	0.003	0.213
Loving duration								0.282	0.308	0.129	0.200
Loving intensity									−0.111	−0.431^*^	−0.053
HAD anxiety										0.213	−0.468^*^
HAD depression											0.163

* *Correlation is significant at the 0.05 level (2-tailed)*.

** *Correlation is significant at the 0.01 level (2-tailed)*.

### Procedure

As in our prior behavioral study (Ortigue et al., 2010b), this study took place over two visits (Visit 1 and Visit 2). Visit 1 was dedicated to select participants on the basis of inclusion-exclusion criteria and to create the stimuli, which involved photographing participants' arm and their significant partner's arm in action, individually. An experimenter (EJ) photographed each partner individually, without letting either one attend the photoshoot of the other partner^1^. At this stage, participants were not explicitly told that these stimuli would be specifically included in the behavioral task. In a separate session, EJ also photographed individuals who were not familiar to any of the participants. To control for distinguishing signs, all of the actors were asked to remove any stigma (e.g., piercing, bracelets, and rings) that could allow their identification. Visit 2 happened a few days after Visit 1 *(M* = 11.2 days, *SD* = 9.17) and was devoted to the completion of the experimental paradigm.

### Experimental paradigm

**Figure 1:**
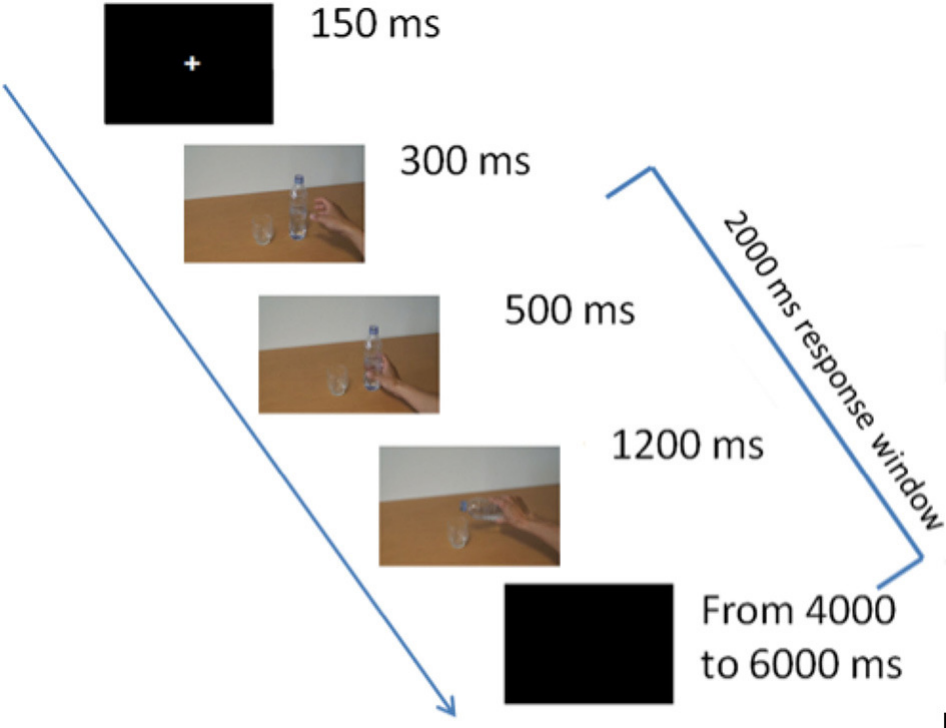
Experimental design. The first 300 ms-frame depicted the agent's right hand reaching one of two objects (e.g., a bottle of water) positioned on an empty table with neutral background. The second 500 ms-frame depicted the agent's right hand grasping that object. The third 1,200 ms-frame displayed the final intention (e.g., filling the glass of water). The three frames appeared immediately following each other, giving the visual illusion of a continuous video-clip. Finally, a 4,000–6,000 ms-inter-trial interval separated the onset of each video-clip presentation.

Figure 1 displays the experimental paradigm. All participants completed one practice block with a few trials of our standard intention inference task (IIT) (Ortigue et al., 2009a,b, 2010b,c; Cacioppo et al., 2014) in order to ensure that they understood the task and instruction. Then, during the full version of the IIT, participants observed a series of sequences of three frames presented centrally on a monitor screen. As in our previous studies (e.g., Ortigue et al., 2010b,c), there was no interval between frames: The first picture/frame displayed the object's scene with the actor's right hand reaching to one (e.g., a bottle of water) of two objects (e.g., a bottle of water and a glass) that were positioned on an empty table with neutral background, and which were presented on screen for 300 ms. The second picture/frame depicted the actor's right hand grasping that object (e.g., the bottle of water) with a specific intent, and was displayed for 500 ms. Finally, the third picture/frame unravels the actor's intention (e.g., filling the glass of water) by showing their right hand completing the action with that object, and was displayed for 1,200 ms. The stimuli were thus presented on screen for a total of 2,000 ms. A total of 54 sequences per block (3 actors × 3 intentions × 6 objects) were presented in each of the four blocks (216 total sequences). In total, four blocks took 50 min to perform, including breaks.

### Participants' instruction

During the task, participants were asked to indicate as rapidly and accurately as possible what the actor's intention was by pressing keys (key 1 for self-oriented actions, 2 for other-oriented actions, and 3 for object oriented actions). Participants had 2 s to respond from the onset of the second frame. As in previous studies (Ortigue et al., 2009a,b, 2010b,c; Decety and Cacioppo, 2012), participants were not explicitly asked to judge category membership of the acting agent (i.e., self, significant other, or stranger).

### Stimuli

Stimuli included photographs of each participant's right forearm reaching for six daily objects (phone, sunglasses, toothbrush, flower, pen, bottle of water) with three different intentions (self-oriented intentions: e.g., grasping a bottle of water to drink; other-oriented intentions: e.g., grasping a bottle of water to offer it to an observer; object-oriented intentions: e.g., grasping a bottle of water to fill a glass). An equal number of each intention type was created to increase the number of stimuli in the IIT. Comparable photographs were taken for a stranger, as well as for the participant's significant other. Similar to our previous study (Ortigue et al., 2010b,c), photographs were taken with a digital camera (Sony Camera HDR-XR550) in a white background with a plain brown table. The camera was placed on a tripod behind the participant's seat in order to capture the right forearm and hand reaching for an object from a first-person perspective. As in previous studies (Hamilton and Grafton, 2007), three pictures were selected to represent three movement phases. As described by Hamilton and Grafton (2007), the advantage of the three-frame video clip is the tight amount of possible kinematic, task duration, and timing control (Hamilton and Grafton, 2007). Stimuli were presented using E-Prime pro (Psychology Software Tools Inc., Sharpsburg, PA, USA) on a computer monitor located 140 cm from the participants (when performed outside the scanner). During the scanning fMRI session, participants viewed the stimuli on a back-projection screen mounted on the head coil of the MRI scanner.

### Behavioral analyses

In line with the hypotheses, we collapsed across intention types and acting hands, yielding a repeated-measures design with agent type (self, significant other, or stranger) as a within-subjects factor. Repeated measures ANOVAs were utilized to analyze potential differences in reaction time and accuracy between agent types in the IIT. Additionally, the relationship between questionnaire data (including measures for cognitive interdependence, as indexed by the IOS scale) and reaction times and accuracy, respectively, was examined through correlational analyses. In order to better understand how reading the actions of a significant partner differed from identifying actions of oneself and whether reading the actions of familiar agents differed from those of unfamiliar agents, we performed orthogonal contrasts examining the behavioral performance in response to the understanding of actions performed by the partner compared to the self: [Partner (+1) > Self (−1)] and those of partner and self, compared to the stranger [(0.5 Self + 0.5 Partner) > Stranger]. Orthogonal contrasts ensured we are not inflating the Type I error rates in the research.

### Magnetic resonance imaging recordings

We recorded MRI data using a 3 T whole-body Trio system (Siemens, Erlangen, Germany), with the standard head coil configuration. For each participant, we acquired structural images using a 3 Dimension-Gradient Recalled Echo (3D-GRE) T1-weighted sequence (field of view [FOV] = 256 mm, TR/TE/Flip = 19 ms/2.27 ms/9°, matrix = 256 × 256, slice-thickness = 1 mm); and functional images with a GRE Echo Planar Imaging (EPI) sequence (relaxation time [TR]/echo time [TE]/Flip = 2,000 ms/30 ms/80°, FOV = 205 mm, matrix = 64 × 64). Functional images were collected continuously, and each functional image comprised 35 contiguous with 3.2 mm axial slices (TR = 2 s) that were parallel to the inferior edge of the occipital and temporal lobes (Cojan et al., 2009).

### Functional image processing and analyses

We, then, analyzed the functional images using the general linear model for event-related designs with the AFNI program 3dDeconvolve (Ward, 2002). For pre-processing, image volumes were co-registered (realigned), corrected for temporal outliers using the AFNI program 3dDespike, spatially smoothed using a 5 mm FWHM Gaussian kernel, and spatially normalized to the MNI atlas template. The general linear model was then used to generate parameter estimates of activity at each voxel, for each experimental condition in each participant. Statistical parametric maps were generated from linear contrasts between the HRF parameter estimates for the different conditions. Voxel time-series were scaled to percent signal change from baseline, so the resulting beta values from the general linear model would correspond to the same percentage change from the baseline estimate. Responses were estimated using the AFNI 3dDeconvolve “tent” basis function model, with responses modeled from zero to 16 s using seven tent functions to generate the impulse response curve within each voxel. In subsequent group analyses, response functions were collapsed to the average of the TR period from 2 to 14 s. In order to identify whether participants' brain activity for understanding the actions of a close romantic partner differed from that sustaining the understanding actions of oneself, and whether participants' brain activity for understanding the actions of familiar agents differed from that sustaining the understanding actions of unfamiliar agents, we performed random-effects group orthogonal contrasts examining participants' brain activity in response to the understanding of actions performed by the partner compared to the self: [Partner (+1) > Self (−1)] and those of partner and self, compared to the stranger (0.5 Self + 0.5 Partner) > Stranger).

Regions showing effects were identified using a cluster analysis with a voxelwise *p*-level of *p* < 0.01. Our results were corrected for multiple comparisons by choosing a cluster size (*k*) of 27 contiguous voxels, connected corner-to-corner (729 μl). This cluster size was determined with a Monte Carlo simulation using the AFNI 3dClustSim program. A volume mask was created from voxels included in the analysis, within which activation was modeled with a voxelwise height threshold of *p* < 0.01 with 5 mm Gaussian FWHM smoothing. The simulation was iterated 10,000 times, resulting in a cluster size of >26 voxels at a corrected alpha < 0.5.

In order to better understand the specific role of the brain areas that were found in the above contrast, we also conducted network analyses [an approach analogous to a Psychophysiological interaction (PPI) analysis], which are useful for determining whether the correlation in activity between two distant brain areas varies under certain conditions, such as different psychological contexts (O'Reilly et al., 2012). This approach allows for search of potential co-activations with these two nodes during intention inferences. A second principle underlying this type of analysis is that the interactions between brain areas potentially change as a function of the psychological context, and that this will be reflected as a change in correlation between the time-courses of those areas (Friston et al., 1997; O'Reilly et al., 2012). Following the group contrast for [(0.5 Self + 0.5 Partner) – Stranger], regions were identified for a seed analysis of contrast-sensitive connectivity in the whole brain to identify voxels showing time-series responses that were correlated with the average time-series of the seed region with respect to the given contrast of interest. Four seed regions of interest were identified from the contrast analysis: Left Inferior Parietal Lobule (IPL), Right Inferior Parietal Lobule (IPL), Left Middle Temporal Gyrus (MTG), and Right Middle Temporal Gyrus (MTG). The analysis was conducted with the same procedure for each seed region: the time-series were averaged across all voxels in the region, giving an average time-series for the seed, baseline TRs (during which no stimuli were presented) were set to zero, TRs during which Self and Partner stimuli were presented were weighted as +1, and periods during which Stranger stimuli were presented were inverted by weighting them as −1. Thus, a contrast-sensitive seed time-series was generated, which was then correlated with the raw time series in each voxel to identify regions showing a significant correlation with the contrast-weighted time series model. Then, using using Fisher's Z transformation, the resulting voxelwise *R*-values were transformed into *Z*-values. The resulting set of values was entered into a group-level voxelwise 1-sample *t*-test to identify voxels wherein the correlations differed from zero. The resulting map of connectivity regions was finally identified using the cluster analysis parameters described above. Finally, correlational analyses were conducted to examine the relationship between BOLD contrasts and questionnaire data.

## Results

### Behavioral results

#### Accuracy

Results revealed a main facilitation effect of the actor on intention understanding [*F*_(2, 46)_ = 3.32, *p* < 0.05, *d* = 0.126], with participants being more accurate at inferring intentions of their partner (*M* = 91.15%, *SD* = 6.68) or of themselves *M* = 89.81%, *SD* = 7.79), compared to intentions of a stranger (*M* = 88.60%, *SD* = 8.13). As expected no difference was observed between the Partner and Self condition [*F*_(1, 23)_ = 2.022, n.s.; *d* = 0.184; Table 3]^1^. Orthogonal planned contrasts also revealed a significant difference between (Partner + Self)/2 and the Stranger [*F*_(1, 23)_ = 4.373, *p* = 0.048; *d* = 0.250]. Correlational analyses yielded no significant results for accuracy and questionnaires, except a positive correlation (*r* = .338; *p* = 0.19) between IOS and partner minus stranger accuracy index, suggesting that the closer the participant felt to their partner the more pronounced was the difference in accuracy between the partner's and the stranger's condition.

**Table 3:** Behavioral results.

**Measure**	**Group**	**Mean (*SD*)^a^**	***n***	***F(df)***	**Significance**	***d***
**ACCURACY**						
	All subjects	89.85% (7.52%)	24	3.32 (2,46)	0.045	
	Stranger vs. (self + partner)/2			4.373 (1,23)	0.048	0.160
	Self vs. partner			2.022 (1,23)	n.s.	0.081
	fMRI subjects	89.77% (4.51%)	12	1.53 (2,22)	n.s.	
**REACTION TIME (Z SCORE)**						
	All subjects	1,254.55 (97.06)	24	0.135 (2,46)	n.s.	
	Stranger vs. (self + partner)/2			0.001 (1,23)	n.s.	0.000
	Self vs. partner			0.339 (1,23)	n.s.	0.015
	fMRI subjects	1,197.39 (63.87)	12	0.835 (2,22)	n.s.	

a *Reported means for reaction time are actual values in ms, not Z scores*.

#### Reaction times

No significant results were observed. Results showed no significant main effect of agent [*F*_(2, 46)_ = 0.135, *p* = 0.874], with reaction times for the Self (*M* = 1254 ms, *SD* = 101.26), Partner (*M* = 1254 ms, *SD* = 92.90), and Stranger (M = 1249 ms, *SD* = 93.28) being not significantly different from one another. Similarly planned contrasts revealed no significant results [(Partner + Self)/2 vs. Stranger: *F*_(1, 23)_ = 0.001, n.s.; *d* = 0.003; Partner vs. Self: *F*_(1, 23)_ = 0.339, n.s.; *d* = 0.046; (Table 3)]. Correlational analyses yielded no significant correlations between reaction times and questionnaires.

### Preliminary neuroimaging results

**Figure 2:**
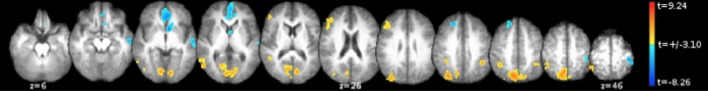
Bold activity obtained for the [0.5 ^*^ Self + 0.5 ^*^ Partner] vs. Stranger contrast. Corrected voxelwise results of the two-tailed *t*-test are shown: regions for which [0.5 ^*^ Self + 0.5 ^*^ Partner] > Stranger are depicted in the red spectrum and Stranger > [0.5 ^*^ Self + 0.5 ^*^ Partner] results are shown in the blue spectrum. These regions are detailed in Tables 4A,B.

In line with our accuracy results, a significant differential activation was observed between the Partner and Stranger (Partner > Stranger contrast) as well as between the Self and Stranger (Self > Stranger), indicating that the two familiar agents showed differential activity as compared to the Stranger, with both contrasts showing significant regions of activity in the regions associated with familiarity (e.g., Yonelinas, 2002; Yonelinas et al., 2005). As expected, no significant differences were observed in the Self > Partner contrast or the Partner > Self contrast. Non-orthogonal contrasts between partner, stranger, and self are displayed in supplementary material (See Supplementary Material). The (0.5 Self + 0.5 Partner) > Stranger contrast also revealed a significant set of brain areas that include (but are not restricted to) brain regions involved in intention understanding within and beyond the inferior fronto-parietal intention network, extending to the middle temporal gyrus (MTG) (see details in Table 4A; Figure 2; Juan et al., 2013,^2^).

**Table 4:** Neuroimaging results.

**Volume (μl)**	**Region(s)**	***x***	***y***	***z***	***t***
**(A) (0.5 SELF + 0.5 PARTNER) > STRANGER**					
6,345	Medial calcarine gyrus	−1	76	5	3.72
	Medial lingual gyrus				
	Medial cuneus				
5,643	Right precuneus	7	68	47	4.18
	Right superior parietal lobule				
2,646	Right middle occipital gyrus	34	76	32	3.84
	Right superior occipital gyrus				
	Right angular gyrus				
1,755	Right middle frontal gyrus	46	−34	22	3.54
	Right inferior frontal gyrus				
1,674	Right inferior parietal lobule	41	45	52	3.66
	Right postcentral gyrus				
	Right superior parietal lobule				
999	Right middle temporal gyrus	55	58	3.5	3.52
756	Left inferior parietal lobule	−38	44	39	3.72
**(B) (0.5 SELF + 0.5 PARTNER) < STRANGER**					
4,698	Medial mid-orbital gyrus	0	−45	−1	−3.75
	Medial rectal gyrus				
	Medial anterior cingulate				
	Medial superior medial gyrus				
1,377	Left middle temporal gyrus	−59	16	−5	−3.65
	Left superior temporal gyrus				
1,053	Right superior frontal gyrus	21	−39	40	−3.50
	Right middle frontal gyrus				
972	Left caudate nucleus	−5.4	−15	−5	−4.10
	Left rectal gyrus				
	Left olfactory cortex				
756	Left postcentral gyrus	−38	30	59	−3.95
	Left precentral gyrus				

**Figure 3:**
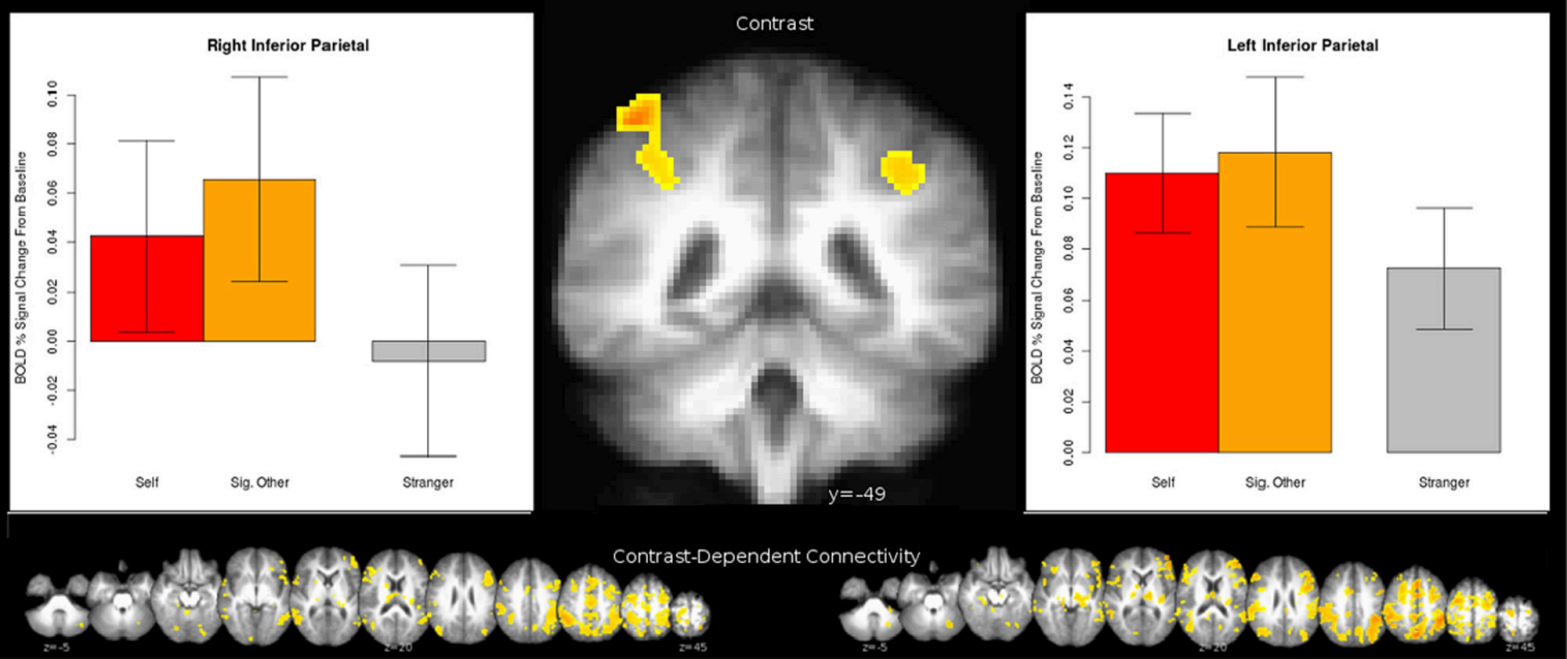
The 0.5 ^*^ Self + 0.5 ^*^ Partner > Stranger effect in the IPL. Clusters were identified from a whole-brain voxelwise analysis (see Figure 2 and Table 4A). Laterality is shown in radiological orientation. Charts designate the mean activity of conditions averaged across all voxels within each respective cluster, with error bars indicating standard error of the mean.

**Figure 4:**
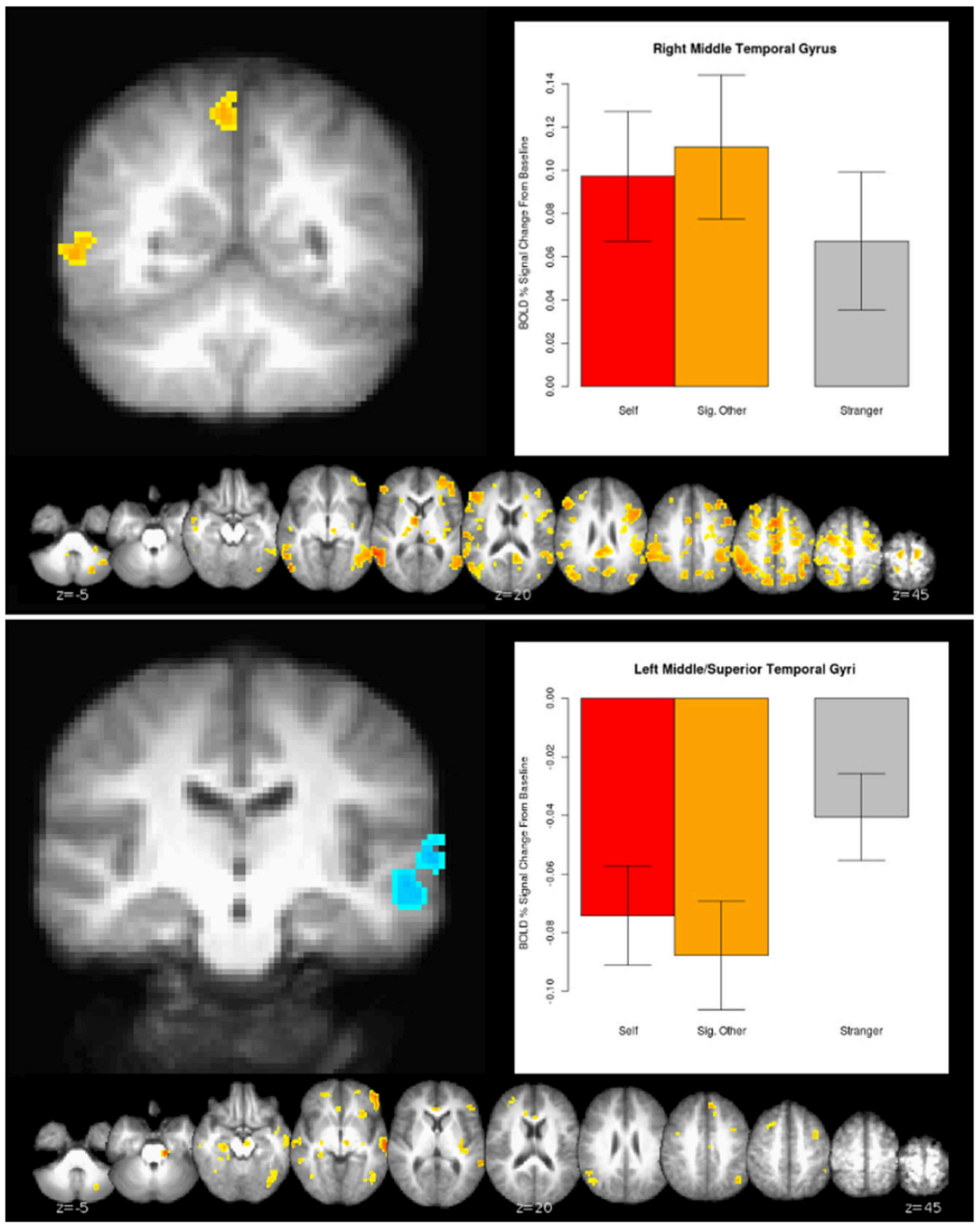
The 0.5^*^Self + 0.5^*^Partner > Stranger effect in the MTG. Clusters were identified from a whole-brain voxelwise analysis (see Figure 2 and Table 4A). Charts designate the mean activity of conditions averaged across all voxels within each cluster, with error bars indicating standard error of the mean.

The connection analyses using IPL as a seed revealed that IPL is connected to many cortical and also subcortical areas, such as the ventral tegmental area (VTA)—a dopaminergic receptor-rich subcortical area that has been previously associated with pair-bond (Cacioppo et al., 2012a,b). See Figure 3 for a broader picture of all the brain areas connected to the left and right IPLs for the (0.5 Self + 0.5 Partner) > Stranger contrast. Similarly, several brain areas appeared to be co-activated when we performed the connection analysis for the right MTG for the same (0.5 Self + 0.5 Partner) > Stranger contrast (Figure 4).

### Correlation analyses

In line with Hebbian principles of neural modulations, a negative correlation between [BOLD [(0.5 ^*^ Self + 0.5 ^*^ Partner) – Stranger] × IOS] was found in several brain regions, such as VTA and BA8), while a positive correlation was observed between [BOLD [(0.5 ^*^ Self + 0.5 ^*^ Partner) – Stranger] × IOS] in other regions, such as the pons and right cerebellum. The negative correlation suggest that the more the participants reported being cognitively close to their partner, the less the brain areas associated with action self-other comparison (e.g., inferior parietal lobule), attention (e.g., superior parietal lobule), recollection (hippocampus), and pair bond (VTA) were recruited, suggesting that the more a shared mental representation has been pre-established, the more neurons show suppression in their response to the presentation of information to which they are sensitive (Barron et al., 2016).

Finally, correlational analyses between self-report questionnaires and the (0.5 Self + 0.5 Partner) > Stranger contrasts also revealed positive correlations between the various self-questionnaires about feelings and brain areas associated with emotions: (1) a positive correlation between [BOLD [(0.5 ^*^ Self + 0.5 ^*^ Partner) – Stranger] × PLS scores] in the left superior temporal gyrus and left insula; (2) a similar positive correlation between [BOLD [(0.5 ^*^ Self + 0.5 ^*^ Partner) – Stranger] × relationship intensity] in the left superior temporal gyrus and left insula; (3) a positive correlation was observed between [BOLD [(0.5 ^*^ Self + 0.5 ^*^ Partner) –Stranger] × Relationship Satisfaction] in RMTG. See Supplementary Material for further details. No clusters were found to be significant for the correlations between BOLD [(0.5 ^*^ Self + 0.5 ^*^ Partner) – Stranger] × Relationship Duration].

## Discussion

Our results reinforce and expand prior research by demonstrating that participants are better at inferring intentions performed by a familiar actor than by a non-familiar actor, and that this better performance was associated with greater activation within and beyond the inferior frontoparietal network i.e., in brain areas related to familiarity (e.g., precuneus). A limitation of the present study is, however, that it included only a small number of participants. Neuroimaging studies with small sample size tend to have low statistical power, reduce the likelihood of detecting a true effect and increase the likelihood of detecting false effects (Button et al., 2013; Cacioppo et al., 2013). Replication with a greater number of subjects will increase our confidence in the generalizability of our preliminary fMRI findings. It is important to note that our confidence in our preliminary results comes from a control contrast that shows similar results to those we obtained in a recent multilevel kernel density fMRI meta-analysis we performed with 306 subjects (Juan et al., 2013). More precisely, in the present study, the brain network involved in understanding intentions performed by an actor (independently of their identity) recruits the expected action observation network (AON), which includes the inferior parietal lobe and inferior frontal gyrus (See Supplementary Material, Juan et al., 2013).

Moreover, our results are consistent with neuroimaging studies on the neural networks sustaining familiarity (Yonelinas et al., 2005; Wang et al., 2010; Wang and Yonelinas, 2012; Wolfe et al., 2017) and person identity (Anzellotti and Caramazza, 2017), as well as models that show an association and common neural bases between familiarity-based recognition and implicit memory (Yonelinas, 2002; Yonelinas et al., 2005 for review; Wang and Yonelinas, 2012). For instance, a large body of studies have shown that the middle/medial temporal lobe play a common role in both familiarity (Yonelinas, 2002 for review) and conceptual implicit memory (e.g., Wang et al., 2010).

Moreover, the present results are line with the working internal predictive model of intention understanding, which suggests that familiarity should act as a facilitating agent due to pre-established, shared mental representations between the two familiar individuals i.e., the observer and the actor. These findings reinforce prior studies that support theories of simulation and embodied cognition by demonstrating that better performance in predicting one's intentions when that the observer is pre-familiar with the actor. Based on our prior study that investigated the role of various types of familiarity on intention understanding (Ortigue et al., 2010b), we are hypothesizing that the present facilitation effect of familiarity on intention understanding also extends beyond basic perceptual familiarity, and rather occurs at a more associative level.

For instance, we found that the inferior parietal lobule and inferior frontal gyrus, two critical nodes of the MNS were specifically recruited for the understanding of intentions performed by the self and partner than for a stranger. We interpret these findings as a involvement of the inferior fronto-parietal network (including the MNS) in interpersonal intention understanding. Moreover, we interpret this mirror-driven facilitation effect as reflecting a shared implicit mental representation of the past actions between the participant and their partner—a hallmark in dyadic relationships, in which actions and intentions of both partners are stored in a “transactive,” intricate web of mental representations that calls for cognitive interdependence. These results provide an account for facilitation effects of embodied cognition and self-other expansion on intention understanding among dyads (Ortigue et al., 2010b,c). The present recruitment of the angular gyrus is in line with prior studies showing its involvement in mental self-related processes and self-other integrative cognitive processes (e.g., Ortigue et al., 2007; Ortigue and Bianchi-Demicheli, 2008). Although our results might be seen as bottom-up attention mechanisms due to the specific implicit salience of the stimuli (Ortigue et al., 2007), this interpretation does not account for all the brain activations. We observed, for instance, brain activations in associative visual processing and self-expansion (angular gyrus; Cacioppo, 2016, 2017). The activation of the angular gyrus during the presentation of familiar agents, as compared with strangers, reflects more elaborate, top-down cognitive processes than bottom-up processes. This assumed top-down influence is in line with the higher-order role of the angular gyrus in conceptual knowledge (e.g., Fairhall and Caramazza, 2013; Seghier, 2013), abstract representation of the self and in significant other processing (Ortigue et al., 2007; Cacioppo, 2016). This suggests that an abstract, integrated representation of a significant familiar agent has been stored along with the representation of one's self in this brain region. Together, these shared self/significant other representations facilitate intention understanding of actions performed by both the self and the significant other.

Consistent with our hypotheses, our results showed a positive correlation between IOS level and partner/stranger accuracy index, suggesting that the closer the participant felt to their partner the more pronounced was the difference in accuracy between the partner's and the stranger's condition (in favor of the partner). Together, our results are in accord with theories suggesting that kinematic cues from biological motion are sufficient to provide implicit information about the person's identity despite the lack in existence of a definite recognition cue for individual identification (Cutting and Kozlowski, 1977; Jokisch et al, 2006; Ortigue et al., 2010b; Cook et al., 2011).

In addition, and in line with Hebbian principles of neural modulations, the more the participants reported being cognitively close to their partner, the less the brain areas associated with action self-other comparison (e.g., inferior parietal lobule), attention (e.g., superior parietal lobule), recollection (hippocampus), and pair bond (VTA) were recruited, suggesting that the more a shared mental representation has been pre-established, the more neurons show suppression in their response to the presentation of information to which they are sensitive. These results suggest that the relation of performance to the extent of neural activation during intention understanding may display differential relationships based on the cognitive domain, brain region, and the cognitive interdependence between the observer and the actor.

Cognitive interdependence between two familiar agents can provide a processing advantage during which each familiar, close partner may implicitly read each other's motor actions and intentions through the automatic reenactment of their shared mental representations—a mechanism that implies that the human brain not only predicts others' mental states and intentions, but also predicts itself. Further studies could be done to take our findings further and investigate different types of familiar close others on their anticipatory behaviors and performance and test the effects of different levels of IOS, while controlling for duration of familiarity. For instance, and based on the theories of simulation and embodied cognition, one may be interested in comparing the effect of a mother's intentions vs. a father's intentions. In one of our studies examining attachment mechanisms in a matrilineal society (society, where the children are raised by both their mother, and their aunt, which then make the face of the mother and the aunt equally familiar to the children; Dai et al., 2014), we showed that the emotional attachment between mother and child has neural ramifications across three successive processing stages of face processing that are distinguished from the neural effects of facial familiarity (Dai et al., 2014). One could imagine testing intention understanding (rather than attachment) in a similar population, and test the facilitation effects of different levels of IOS for the mother vs. an aunt on intention understanding, while controlling for duration of familiarity.

## Ethics statement

This study was carried out in accordance with the recommendations of Institutional Review Board of the Faculty of Psychology and Educational Sciences, at the University of Geneva, Switzerland with written informed consent from all subjects. The protocol was approved by the Institutional Review Board of the Faculty of Psychology and Educational Sciences, at the University of Geneva, Switzerland.

## Author contributions

SC formulated hypotheses and designed experimental paradigm; EJ collected data under SC's supervision; SC and EJ analyzed behavioral data. SC and GM performed fMRI analyses; SC wrote the manuscript.

### Conflict of interest statement

The authors declare that the research was conducted in the absence of any commercial or financial relationships that could be construed as a potential conflict of interest. The reviewer PT and handling Editor declared their shared affiliation, and the handling Editor states that the process met the standards of a fair and objective review.

## Footnotes

^1^In no instances did both partners in any given dyad participated in the current study.

^2^On the other hand, the contrast (0.5 Self + 0.5 Partner) < Stranger contrast revealed brain activations within the Social Network (see Table 4B), which involves regions such as the right middle frontal gyrus and the left middle temporal gyrus, both of which are related to biological motion, animacy, affect processing, and theory of mind attribution Grafton, 2009.
