# Supplementary material for: Predicting Intentions of a Familiar Significant Other Beyond the Mirror Neuron System
Source: Front Behav Neurosci. 2017 Aug 25;11:155. doi: 10.3389/fnbeh.2017.00155 (PMC5574908; doi:10.3389/fnbeh.2017.00155)
Supplement: Supplementary file 1 [file Table1.PDF]

Supplementary Material

Table 1

BOLD [(.5*Self+.5*Partner)-Stranger] x Inclusion of the Other in the Self (IOS), p<.01

Index	[SelfPartner-Stranger] x IOS, p<.01	Vol (ul)	x	y	z	R
1	15.2 % overlap with Ventral Tegmental Area, code 40	2916	7.3	-26.2	-11	-0.85
	6.0 % overlap with Right Hippocampus, code 38					
	4.5 % overlap with Cerebellar Vermis, code 110					
2	0.4 % overlap with Pons area, code 116	1323	6.6	-24.6	-38.1	0.83
3	9.9 % overlap with Left Cerebellum, code 107	1161	-20.3	-36.3	-41.6	0.87
	7.9 % overlap with Left Cerebellum, code 97					
	7.1 % overlap with Left Cerebellum, code 105					
	7.0 % overlap with Left Cerebellum, code 103					
4	94.9 % overlap with Frontal Eye Field (BA8), code 7	1080	-35	20.1	50.8	-0.87
	0.4 % overlap with Left Superior Frontal Gyrus, code 3					
5	25.8 % overlap with Right Postcentral Gyrus, code 58	918	21.9	-47.5	47.9	-0.88
	4.3 % overlap with Right Superior Parietal Lobule, code 60					
	3.6 % overlap with Right Inferior Parietal Lobule, code 62					
6	59.1 % overlap with Right Cerebellum, code 104	864	14.3	-60.2	-44.1	0.91
	11.6 % overlap with Right Cerebellum, code 100					
	6.8 % overlap with Right Cerebellum, code 102					
