# Supplementary material for: Predicting Intentions of a Familiar Significant Other Beyond the Mirror Neuron System
Source: Front Behav Neurosci. 2017 Aug 25;11:155. doi: 10.3389/fnbeh.2017.00155 (PMC5574908; doi:10.3389/fnbeh.2017.00155)

Supplementary Material

Figure 1

BOLD [(.5*Self+.5*Partner)-Stranger] x Inclusion of the Other in the Self (IOS), p<.01

C1

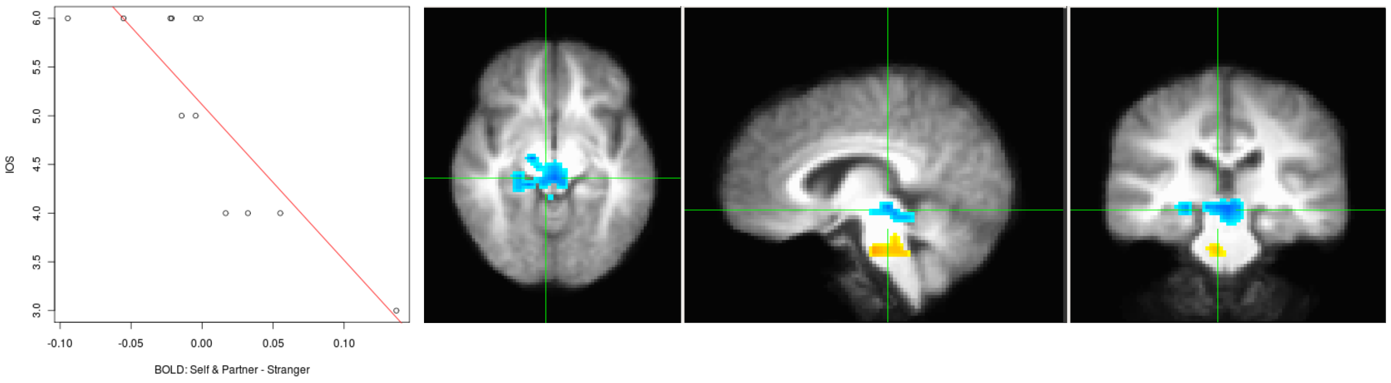

C2

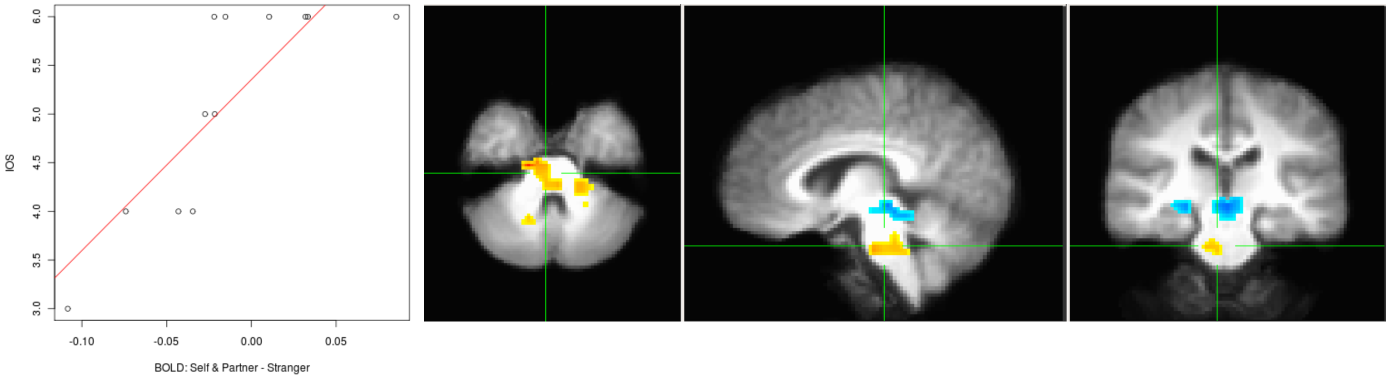

C3

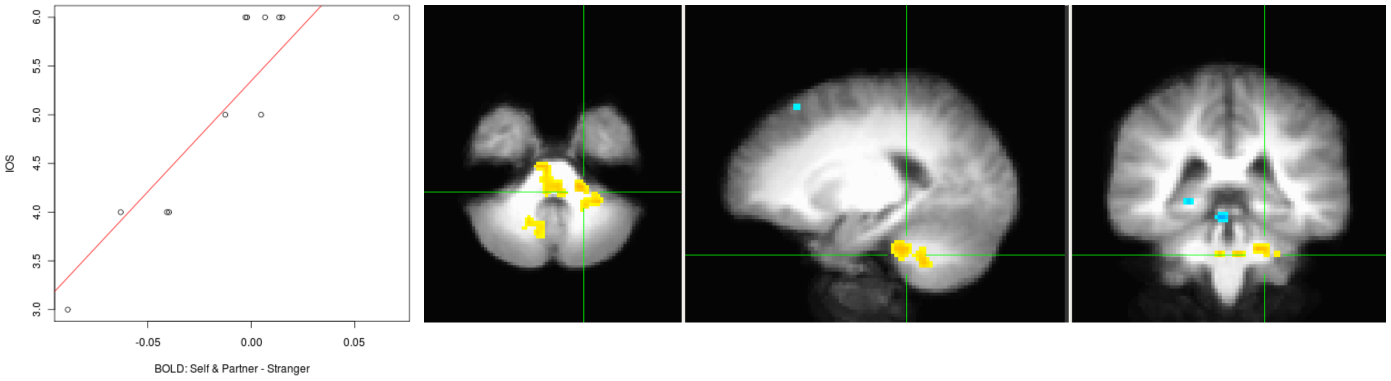

BOLD [(.5*Self+.5*Partner)-Stranger] x Inclusion of the Other in the Self (IOS), p<.01

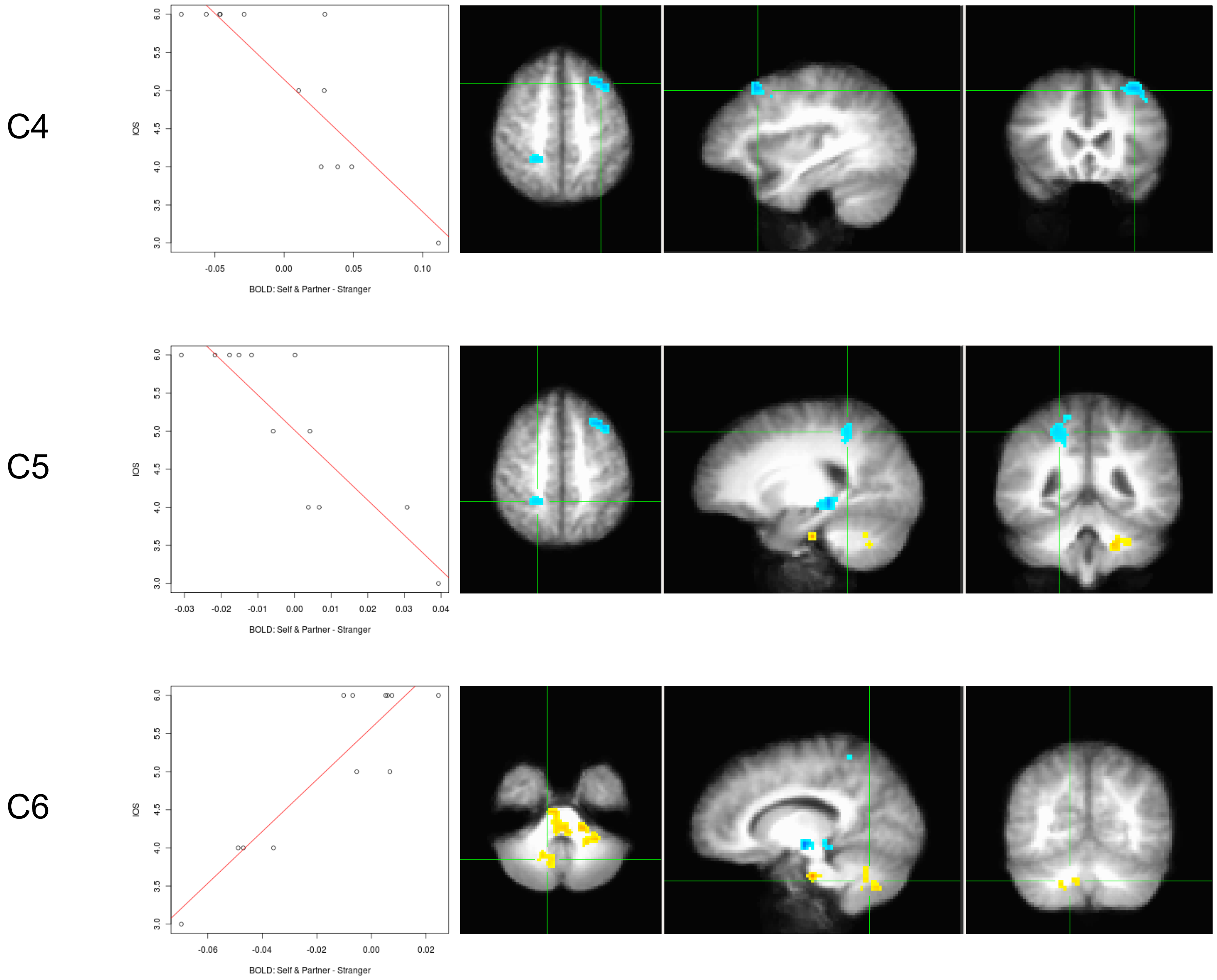

Supplement: Supplementary file 2 [file Image1.PDF]
