# Supplementary material for: Predicting Intentions of a Familiar Significant Other Beyond the Mirror Neuron System
Source: Front Behav Neurosci. 2017 Aug 25;11:155. doi: 10.3389/fnbeh.2017.00155 (PMC5574908; doi:10.3389/fnbeh.2017.00155)
Supplement: Supplementary file 3 [file Image2.PDF]

Supplementary Material

Figure 2

BOLD [(.5*Self+.5*Partner)-Stranger] x Passionate Love Scale, p<.01

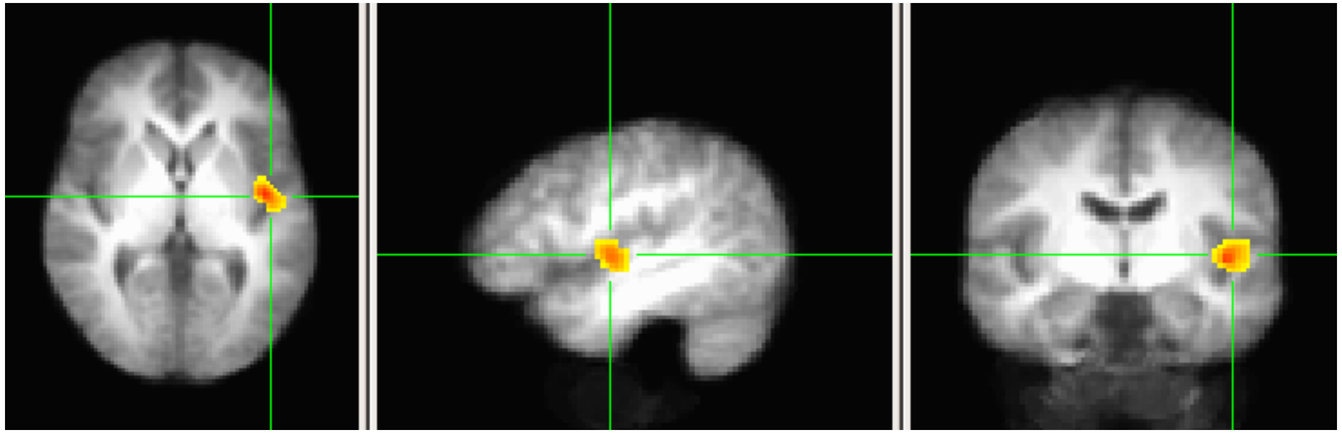

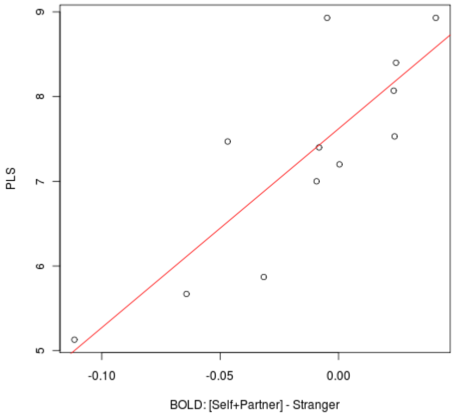

[Self + Partner] – Stranger x PLS	Vol(ul)	x	y	z
65.7 % overlap with Left Superior Temporal Gyrus, code 81	999	-45	-7	2
23.6 % overlap with Left Insula Lobe, code 29				
9.1 % overlap with Left Rolandic Operculum, code 17				
