# Supplementary figures and images for: Predicting Intentions of a Familiar Significant Other Beyond the Mirror Neuron System
Source: Front Behav Neurosci. 2017 Aug 25;11:155. doi: 10.3389/fnbeh.2017.00155 (PMC5574908; doi:10.3389/fnbeh.2017.00155)

# Supplementary Material

Figure 3

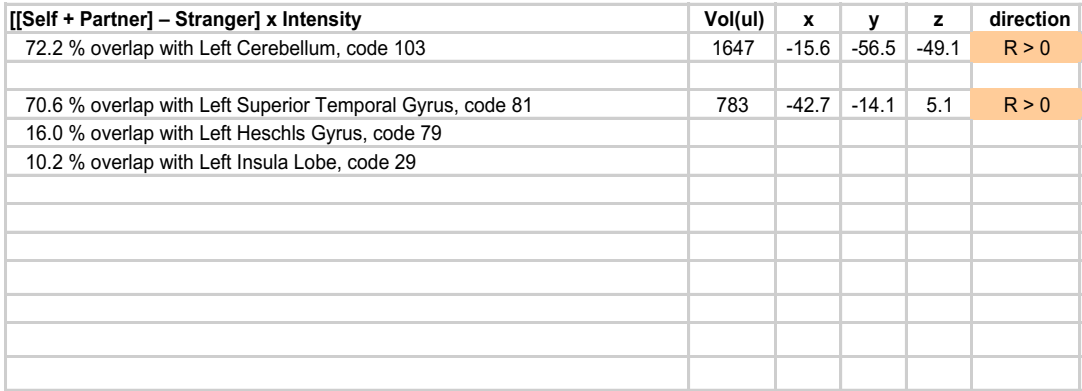

Supplement: Supplementary file 4 [file Image3.PDF]
