# Supplementary material for: Predicting Intentions of a Familiar Significant Other Beyond the Mirror Neuron System
Source: Front Behav Neurosci. 2017 Aug 25;11:155. doi: 10.3389/fnbeh.2017.00155 (PMC5574908; doi:10.3389/fnbeh.2017.00155)
Supplement: Supplementary file 5 [file Image4.PDF]

Supplementary Material

Figure 4

BOLD [(.5*Self+.5*Partner)-Stranger] x Relationship Satisfaction, p<.01

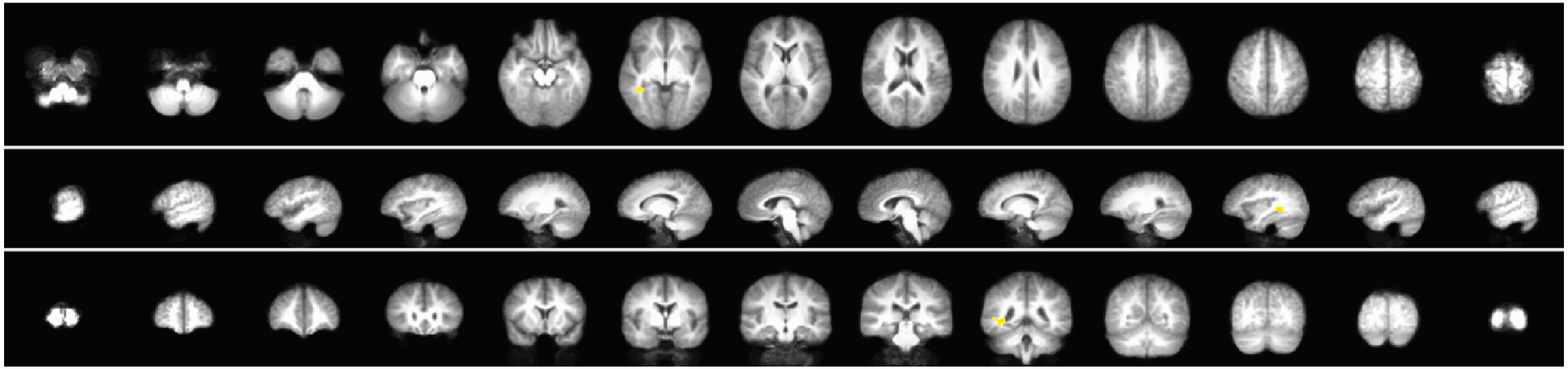

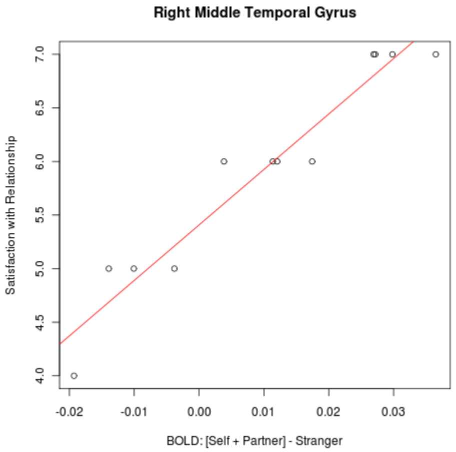

[[Self + Partner] – Stranger] x Satisfaction	Vol(ul)	x	y	z	direction
4.5 % overlap with Right Middle Temporal Gyrus, code 86	756	39.1	-40.4	-2.9	R > 0
