# Supplementary material for: Predicting Intentions of a Familiar Significant Other Beyond the Mirror Neuron System
Source: Front Behav Neurosci. 2017 Aug 25;11:155. doi: 10.3389/fnbeh.2017.00155 (PMC5574908; doi:10.3389/fnbeh.2017.00155)
Supplement: Supplementary file 6 [file Image5.PDF]

# Supplementary Material

Table 2

# BOLD [(.5*Self+.5*Partner)-Stranger] x Relationship Time, p<.01

| Index | [SelfPartner-Stranger] BOLD x Relationship Time, p<.01 | Vol (ul) | x | y | z | R |
|---|---|---|---|---|---|---|
| | ** NO CLUSTERS FOUND *** | | | | | |
