# Supplementary material for: Predicting Intentions of a Familiar Significant Other Beyond the Mirror Neuron System
Source: Front Behav Neurosci. 2017 Aug 25;11:155. doi: 10.3389/fnbeh.2017.00155 (PMC5574908; doi:10.3389/fnbeh.2017.00155)
Supplement: Supplementary file 7 [file Image6.PDF]

BOLD [Intention Understanding – Baseline], p<.01 corrected

Region	Volume (ul)	x	y	z	t
Left Inferior Parietal Lobule	4266	-37.9	-40.9	40.8	4.26
Left Supramarginal Gyrus					
Left Postcentral Gyrus					
Left Lentiform Nucleus	3618	-23.7	1.6	6.8	3.94
Right Middle Temporal Gyrus	3267	49.2	-66	-2.5	4.40
Right Middle Occipital Gyrus					
Right Inferior Temporal Gyrus					
Left Middle Temporal Gyrus	1539	-44.2	-68.2	2.4	4.15
Left Middle Occipital Gyrus					
Left Inferior Temporal Gyrus					
Left Insula	1431	-33.1	19.3	6.4	3.75
Left Inferior Frontal Gyrus					
Right Cerebellum	1188	20.9	-43.9	-29.1	3.69
Right Insula	1080	32.7	18.8	6.1	3.46
Right Inferior Frontal Gyrus					
Left Medial Frontal Gyrus	1080	-7.3	4.6	53.1	3.84
Left Superior Frontal Gyrus					
